# Renal metastasis of gastric cancer caused acute kidney injury which resulted with hemodialysis: case report and literature review

**DOI:** 10.3389/fonc.2024.1459470

**Published:** 2024-08-29

**Authors:** Ivo Dilber, Stjepko Pleština, Domina Kekez, Ivana Vukovac Šokec, Marijana Ćorić, Juraj Prejac

**Affiliations:** ^1^ Department of Oncology and Nuclear Medicine, Zadar General Hospital, Zadar, Croatia; ^2^ Department of Oncology, University Hospital Centre Zagreb, Zagreb, Croatia; ^3^ University of Zagreb School of Medicine, Zagreb, Croatia; ^4^ University of Zagreb School of Dental Medicine, Zagreb, Croatia; ^5^ Department of Internal Medicine, Koprivnica General Hospital, Koprivnica, Croatia; ^6^ Department of Pathology and Cytology, University Hospital Centre Zagreb, Zagreb, Croatia

**Keywords:** gastric cancer, renal metastasis, acute renal insufficiency, kidney biopsy, hemodialysis

## Abstract

Gastric cancer ranks fourth among the most commonly diagnosed cancers, with over a million new cases diagnosed worldwide each year. Acute and chronic kidney damage are common in patients with malignant diseases and are associated with increased risk of complications and mortality. Rarely, acute renal insufficiency may result from bilateral infiltration of renal parenchyma by tumor cells from another organ. We present a case of a patient with clinical suspected gastric cancer and metastases to the kidneys leading to acute renal failure requiring hemodialysis. Despite gastric biopsies, no tumor cells were found, while histopathological examination of enlarged intra-abdominal lymph node biopsy material confirmed adenocarcinoma of signet ring cell originating from the digestive system. Stomach cancer was identified as the most likely primary site after the kidney biopsy was performed. To the best of our knowledge, no case of gastric cancer leading to kidney metastases and acute renal failure requiring renal replacement therapy was yet described. Multidisciplinary collaboration among oncologists, urologists, radiologists, pathologists, and nephrologists is essential for the optimal treatment outcome of these patients, who generally have a poor prognosis.

## Introduction

Gastric cancer ranks fourth among the most commonly diagnosed cancers, with over a million new cases diagnosed worldwide each year (1). At the time of diagnosis, most patients are in an advanced stage of the disease due to nonspecific symptoms of the earlier stages and the absence of early screening, except in East Asian countries (2, 3). Adjuvant treatment for gastric cancer consists of chemotherapy or chemoradiotherapy depending on the type of previous surgery. Combination therapy with platinum salts (oxaliplatin or cisplatin) and fluoropyrimidine preparation (fluorouracil or capecitabine) is used for the treatment of metastatic disease, with the addition of trastuzumab, pembrolizumab, or nivolumab depending on human epidermal growth factor receptor 2 (HER-2) and programmed death-ligand 1 (PD-L1) status. For high microsatellite instability (MSI-H)/mismatch repair deficient (dMMR) tumors, regardless of PD-L1 status, monotherapy with pembrolizumab, dostarlimab, nivolumab with ipilimumab is used (1, 4). Acute kidney failure is defined as a sudden decrease in kidney function that occurs within 7 days, manifested by an increase in serum creatinine concentration and/or reduced urine excretion (5). Acute kidney injury occurs in approximately 10-15% of hospitalized patients and is more common in patients with malignant disease than in the general population (6, 7). The risk factors for developing acute kidney injury in cancer patients include previous chronic kidney disease, diabetes, use of ACE inhibitors or angiotensin receptor blockers, as well as the use of nephrotoxic drugs, chemotherapy, antibiotics, and nonsteroidal anti-inflammatory drugs (6, 8). The etiology of intrinsic kidney damage in this population is broad and can be caused by the toxicity of oncological therapy, metabolic, and immunological factors (9). The most common malignant diseases associated with acute kidney injury are multiple myeloma, renal cell carcinoma, bladder cancer, lymphomas, and leukemias (6, 10). Herein, we present a case of suspected gastric cancer with metastases to the kidneys leading to acute renal failure requiring hemodialysis.

## Case presentation

**Figure 1:**
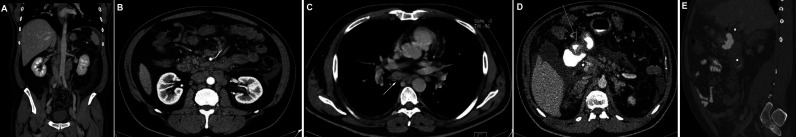
Computerized tomography shows intra-abdominal and hilar lymphadenopathy and no evidence of disease on kidneys **(A-C)** contrast filling defect at the transition of the pylorus to the duodenal bulb **(D, E)**.

**Figure 2:**
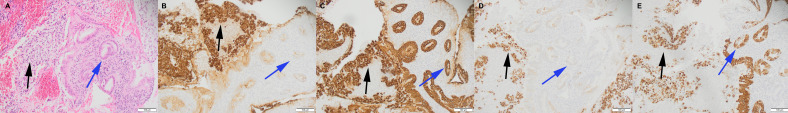
Intra-abdominal lymph node fine-needle aspiration showing atypical cells (signet ring) infiltration in lymph node (Hematoxylin and Eosin magnification ×20) **(A)**. Positive staining for CEA (magnification ×20) **(B)**, CKAE1AE3 (magnification ×20) **(C)**, CK7 (magnification ×20) **(D)**, CK20 (magnification ×20) **(E)** in tumor cells from intra-abdominal lymph node fine-needle aspiration. Black point bar showing tumor tissue. Blue point bar showing normal tissue.

**Figure 3:**
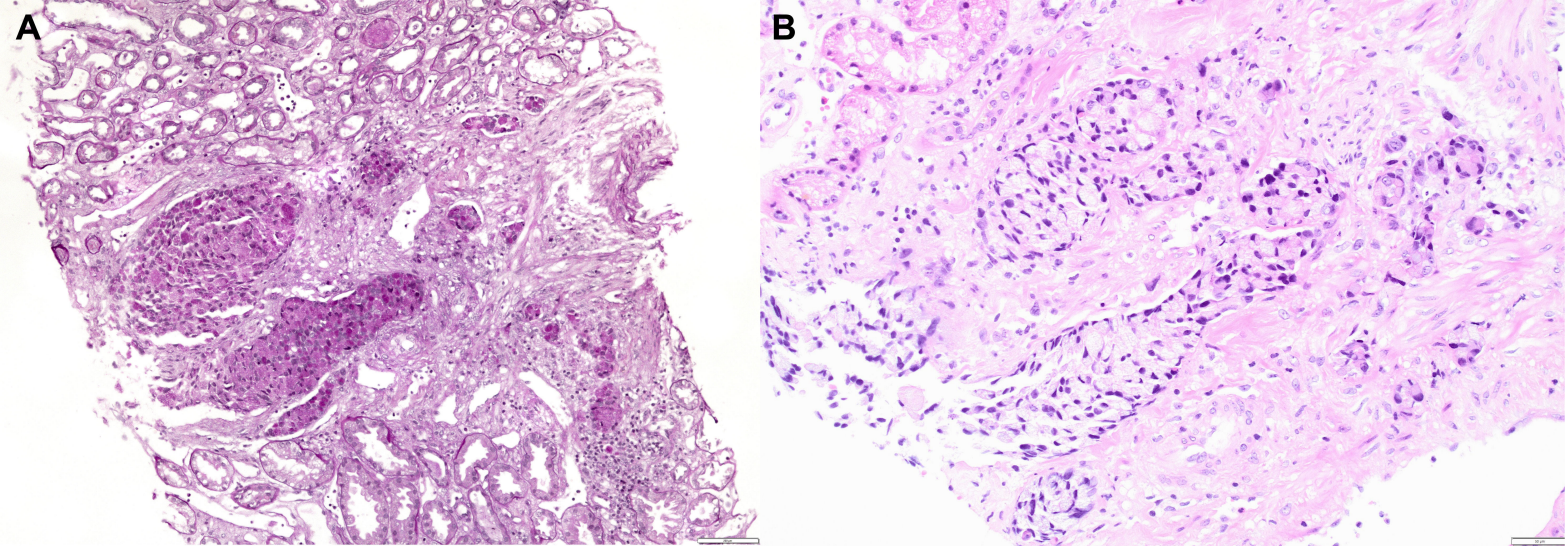
Kidney biopsy showing infiltration of renal parenchyma by PAS positive tumor cells (PAS original magnification ×100) **(A)** and by signet ring tumor cells (H&E, original magnification ×200) **(B)**.

**Figure 4:**
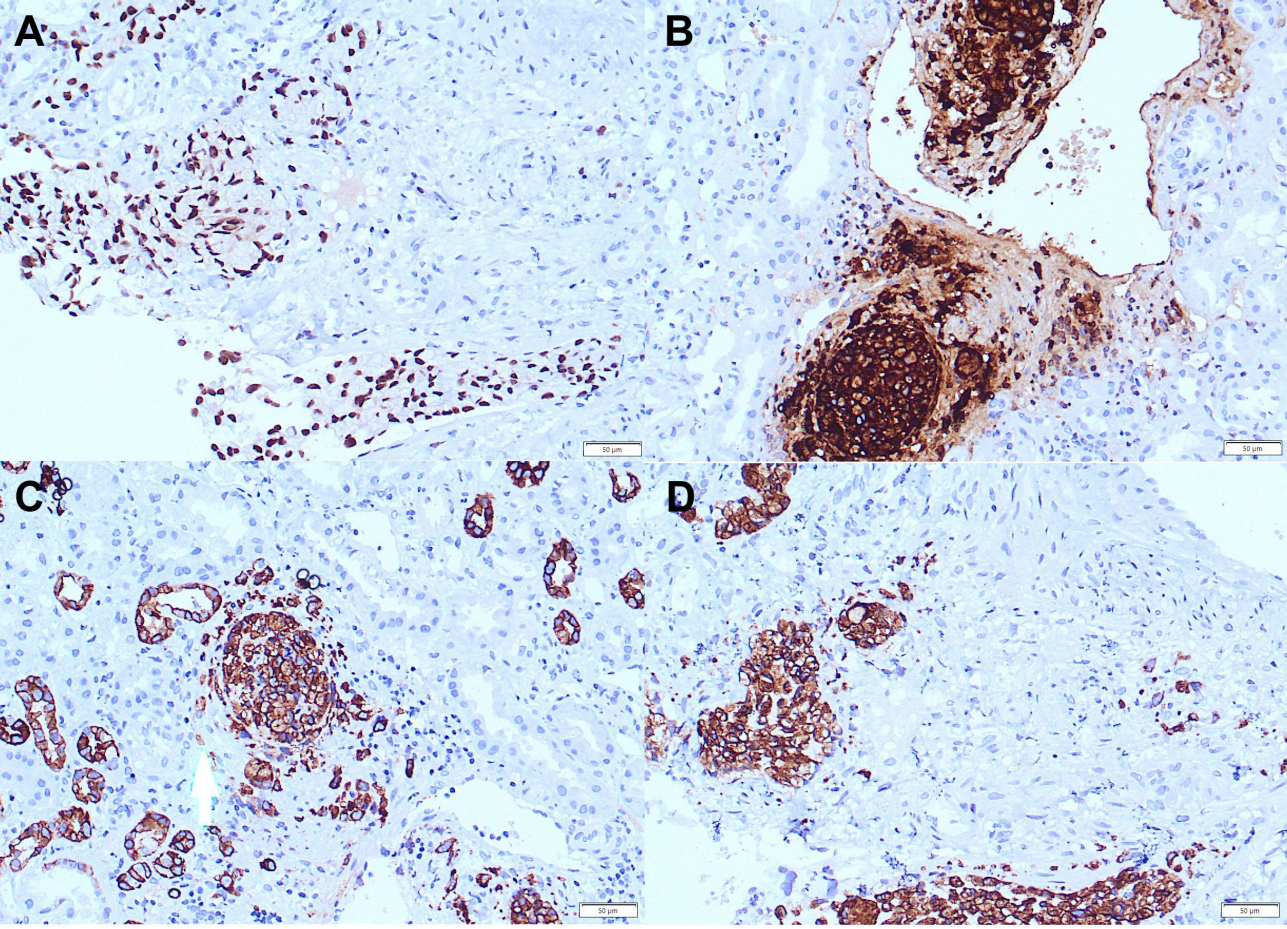
Positive staining for CDX-2 (magnification ×20) **(A)**, CEA (magnification ×20) **(B)**, CK7 (magnification ×20) **(C)**, CK20 (magnification ×20) **(D)** in tumor cells in kidney biopsy.

A 46-year-old Caucasian European man with no history of previous disease was admitted to the hospital due to abdominal pain and weight loss in November 2020. Except for the abdominal pain and tenderness, he presented with no other symptoms and the clinical examination showed no abnormalities besides previously stated abdominal tenderness. No enlarged lymph nodes during the physical examination were found. He had negative family history of malignant disease. The computerized tomography (CT) was performed 2 days after the admission to the hospital and described intra-abdominal and hilar lymphadenopathy and an infiltrative and stenosing process extending from the pylorus to the duodenal bulb at a length of about 4 cm (**Figure 1**). Colonoscopy, wich was performed during the hospitalization, showed no evidence of disease. Gastroscopy showed irregular and thickened stomach lining highly suspected of gastric cancer. Carcinoembryonic antigen (CEA) and Ca 72.4 tumor markers were elevated. CEA was 180 ng/ml (reference value <3.6 ng/ml), while Ca 72.4 was >600 U/L (reference value <6.9 U/L). Other laboratory findings were regular, including kidney ratings (creatinine 97 umol/L, urea 6.8 mmol/L, Leucocytes 9.5 x10e9/L, Erythrocytes 4.67 x10e12/L, Hemoglobin 146 g/L). Repeated endoscopy failed to confirm the diagnosis of malignant disease. The biopsy of the antrum of the stomach showed abundant inflammatory infiltrates of lymphocytes and plasma cells, with occasional granulocytes. The epithelium showed reparative changes of the cells and presence of Helicobacter pylori was positive (+++). Only the endoscopic ultrasound fine-needle aspiration (EUS-FNA) confirmed adenocarcinoma of signet ring cell originating from the digestive system (**Figure 2**). Immunohistochemistry (IHC) tumor was positive to cytokeratin (CK) 7, CK20, CKAE1AE3, carcinoembryonic antigen (CEA), and negative to thyroid trancription factor 1(TTF1) and littoral cell angioma (LCA) (**Figure 2**). He was released from the hospital after 11 days stay. On November 17^th,^ 7 days after the hospital release he was admitted again because of the obstructive jaundice caused by extraluminal compression of the common bile duct by enlarged lymph nodes. Blood test showed bilirubin levels of 241 umol/l (reference value 3-20 umol/L), aspartate transferase-AST 173 U/L (reference value 10-40), alanine-transaminase-ALT 75 U/L (reference value 10-41 U/L), gamma-glutamyltransferase-GGT 137 U/L (reference value 11-60 U/L), alkaline phosphatase 585 U/L, (reference value 40-130 U/L). The endoscopic retrograde cholangiopancreatography (ERCP) was performed with a biliary stent placement which ultimately resolved the jaundice. Patient was released from the hospital and start of chemotherapy treatment was planned. One month after 2^nd^ admission to the hospital, in December 2020 the patient developed acute renal failure with development of oliguria, which required hemodialysis treatment. Creatinine levels in the blood were 892 umol/L (reference value 64-104 umol/L), urea 30 mmol/L (reference value 2.8-8.3 mmol/L) with regular ranges of potassium and sodium. Considering the planned platinum-based chemotherapy protocol, which was not yet given, and due to the suspicion of acute tubulointerstitial damage due to long-term and numerous analgesic therapies, a kidney biopsy was performed. The histopathological evaluation revealed mild acute tubular and interstitial damage with tumor thrombi and micrometastasis in the kidney medulla, while there were no glomerular deposits and negative immunofluorescence (**Figure 3**). Immunohistochemistry (IHC) tumor was positive to CK7, CK20, CEA and CDX-2 (**Figure 4**). Stomach cancer was identified as the most likely primary site. Unfortunately, despite hemodialysis treatment and other symptomatic and supportive care, rapid deterioration of general condition of our patient with worsening of abdominal pain soon followed, which is why it was not possible to start specific oncological treatment, and the outcome was fatal. Our patient passed away two months after his first contact with physician.

## Discussion

Acute and chronic kidney damage are common in patients with malignant diseases and are associated with an increased risk of complications and mortality (6, 10). According to research by Christiansen et al. in Denmark, 17.5% of patients with malignant diseases developed some form of acute kidney failure within the first year, while within 5 years of diagnosis, this percentage reached 27% (6). Acute kidney damage associated with malignant disease can be caused by prerenal, renal, and postrenal damage. The etiology of renal damage in this population is broad and can be caused by the toxicity of oncological therapy, especially platinum-based chemotherapy, metabolic causes such as tumor lysis syndrome and hypercalcemia, immunological causes such as glomerulonephritis and vasculitis, and tubular obstruction such as cast nephropathy (9). Rarely, acute renal insufficiency may result from bilateral infiltration of renal parenchyma by tumor cells from another organ (9). The most common malignant diseases associated with acute kidney damage are multiple myeloma, renal cell carcinoma, bladder cancer, lymphomas, and leukemias (6, 10). Although lymphomas and leukemias are the most common malignant diseases leading to kidney infiltration, they rarely lead to kidney failure (9). Among solid tumors, the most common to metastasize to the kidney are lung cancer, gastrointestinal tumors (colorectal, stomach, esophagus), breast cancer, and melanoma (6). The prevalence of renal metastases in autopsy specimens of patients with solid tumors ranges from 2.36 to 12.6% (11). It is known that gastric tumors metastasize hematogenously, lymphogenously and transperitoneally (12). Tumors from different parts of the stomach likely metastasize in different ways and to different organs due to the uneven blood supply. For example, tumors of the cardia more often metastasize to the lungs because of the direct blood flow from the proximal stomach to the lungs, bypassing the flow through the liver (13). Renal metastases usually manifest as bilateral, small, multifocal parenchymal nodules, but sometimes they can be solitary lesions, posing a differential diagnostic problem with primary renal neoplasms (6, 11). Most cases are subclinical, while some patients experience lumbar pain, hematuria, weight loss, or newly onset or poorly controlled previously known hypertension (6, 8, 14). Acute kidney injury caused by malignant disease infiltration results from destruction of the renal parenchyma, leading to disruption of glomerular, tubulointerstitial and microvascular architecture and function (8, 14). Kidney biopsy remains the gold standard for diagnosing kidney failure in this group of patients. The sensitivity of CT in diagnosing renal metastases is not high, approximately 45.5% (15). Since most data on kidney metastases are based on case reports, there are no clear guidelines for treating solitary kidney metastases, unlike guidelines for treating primary kidney tumors. Surgical treatment of tumor metastases may be an option for selected patients to reduce tumor mass and alleviate symptoms such as pain or bleeding. However, the lack of kidney function recovery due to kidney metastases is a limiting factor for chemotherapy and the use of contrast agents for therapy evaluation. Early diagnosis and removal of the cause or treatment of kidney damage are crucial for kidney function recovery and continuation of oncological treatment. Tugba Yavuzsen et al. described a case of acute renal insufficiency with rapidly progressive glomerulonephritis treated with corticosteroids and hemodialysis. Subsequent examination confirmed gastric adenocarcinoma, which was then surgically removed. Since the patient was in remission after treatment of acute kidney failure and gastric carcinoma surgery, acute kidney failure in this patient was considered a paraneoplastic syndrome (16). Nobuyuki Kaiwara et al. described a case of a patient with confirmed membranous nephritis as the cause of nephrotic syndrome. Further diagnostic workup confirmed gastric adenocarcinoma. After surgery and adjuvant chemotherapy, there were no signs of nephrotic syndrome. Seven years after the end of oncological treatment, the patient again developed nephrotic syndrome, and peritoneal metastases of gastric carcinoma were confirmed several years later (17). Dussol B et al. described a case report of rapidly progressive glomerulonephritis in a patient diagnosed with gastric adenocarcinoma. After treatment with corticosteroids, plasmapheresis, and gastric carcinoma surgery, partial recovery of kidney function was achieved (18). Miyamata et al. described the first case of lung cancer with metastases to the kidneys leading to acute renal insufficiency requiring permanent and successful hemodialysis treatment (19). In the study by Akimoto N et al., a case of an 81-year-old patient diagnosed with gastric cancer is described. The postoperative histopathological findings confirmed adenocarcinoma, stage IIIB. The patient refused adjuvant chemotherapy. Seven months after the surgery, a hypovascular lesion measuring 20 mm was observed on an MSCT scan. Four months later, the tumor had progressed with new metastases in the paraaortic lymph nodes, leading to a right partial nephrectomy. Histopathological findings indicated metastasis of gastric adenocarcinoma. Due to lumbar pain, the patient underwent palliative radiotherapy, resulting in a good clinical response. According to the authors, gastric metastases to the kidneys are very rare but respond well to palliative radiotherapy (20). Unlike our patient, this case involved a solitary metastasis visible on MSCT. The mentioned metastasis did not negatively affect the kidney function of the patient. Reviewing the literature, we did not come across a case description of gastric cancer leading to kidney metastases and acute renal failure requiring renal replacement therapy. In all described cases, the cause of acute kidney damage requiring hemodialysis treatment was mostly part of a paraneoplastic syndrome or rarely due to postrenal obstruction by the tumor. Therefore, the interest of our case is reflected in several aspects. Our patient was significantly younger than expected for a diagnosis of suggested metastatic gastric cancer, as can be seen in hereditary causes. Signet ring cell cancer may arise in younger age and can be found in other tumor sites, but the patient had a normal colonoscopy, as well as CT scan showing normal pancreatic appearance with normal Ca19-9 values, as well as the negative family history of malignant diseases. Clinical and radiological findings (MSCT, endoscopy, tumor marker specific for the stomach) referred to gastric cancer even though the malignancy was not confirmed by biopsy of the gastric lesion but from intra-abdominal lymph node. During our clinical work-up we had incidences of negative biopsies of gastric cancer, which was only later successfully confirmed. Histopathological examination of intra-abdominal lymph node biopsy material done in Zadar General Hospital could not definitively determine the localization of the primary tumor but it suggested that it originated from digestive system. Histopathological examination of kidney biopsy from University Hospital Center Zagreb established gastric cancer as most likely diagnosis although acute kidney failure was initially suspected due to analgesic therapy. There were no clinical, laboratory and histopathological findings that would point to paraneoplastic syndrome or glomerulonephritis. To our opinion, acute kidney failure was developed due to micrometastasis and tumor thrombi in the kidney medulla. Radiological methods did not confirm metastatic changes in the kidneys despite renal insufficiency. Apart from metastases to lymph nodes, the patient had no metastases to other organs, which is present in majority of described cases with presentation of renal metastases. Since there were metastases in intra-abdominal and intrathoracic lymph nodes, filled lymphovascular spaces in renal biopses and tumor thrombi in blood vessels, we believe this indicates lymphogenous and hematogenous metastasis.

In conclusion, this is the first case report, to our knowledge, where gastric carcinoma metastases to the kidneys led to acute kidney injury requiring hemodialysis treatment. Although metastases from malignant diseases rarely cause acute kidney failure, it is important to consider this entity differentially, especially in cases where radiological methods do not describe changes in the kidneys that suggest metastases. With this case report, we want to emphasize the importance of each individual link in the multidisciplinary team in making an accurate diagnosis and treatment decision for oncological patients. In patients with kidney metastases, the multidisciplinary approach of oncologists, urologists, radiologists, pathologists, and nephrologists is essential for the most optimal treatment outcome for these patients, as kidney failure carries an increased risk of complications and mortality. Considering that this is a case report, further research on this topic is necessary. Despite the unusual presentation of the disease, we believe that the decision regarding the choice of treatment for such patients, as well as the monitoring of their response to therapy, should be conducted based on current guidelines for the treatment of metastatic gastric adenocarcinoma.

## Data Availability

The original contributions presented in the study are included in the article. Further inquiries can be directed to the corresponding author.
